# Preparation of TEMPO-Oxidized Cellulose Hydrogels Modified with β-Cyclodextrin and κ-Carrageenan for Potential Adsorption Applications

**DOI:** 10.1021/acsomega.4c08188

**Published:** 2024-12-28

**Authors:** Liliane Oliveira Mota, Yslaine Andrade de Almeida, Diego Fonseca Bispo, Marcos Fabio Farias Souza, Douglas Costa Santos, Raimundo Alves Lima Sobrinho, Iara F. Gimenez

**Affiliations:** † Department of Materials Science and Engineering, Federal University of Sergipe, 49400-000 São Cristóvão, SE, Brazil; ‡ Department of Chemistry, Federal University of Sergipe, 49400-000 São Cristóvão, SE, Brazil; § Department of Chemical Engineering, Federal University of Sergipe, 49400-000 São Cristóvão, SE, Brazil; ∥ Department of Chemical Engineering, State University of Santa Cruz, 45662-900 Ilhéus, BA, Brazil

## Abstract

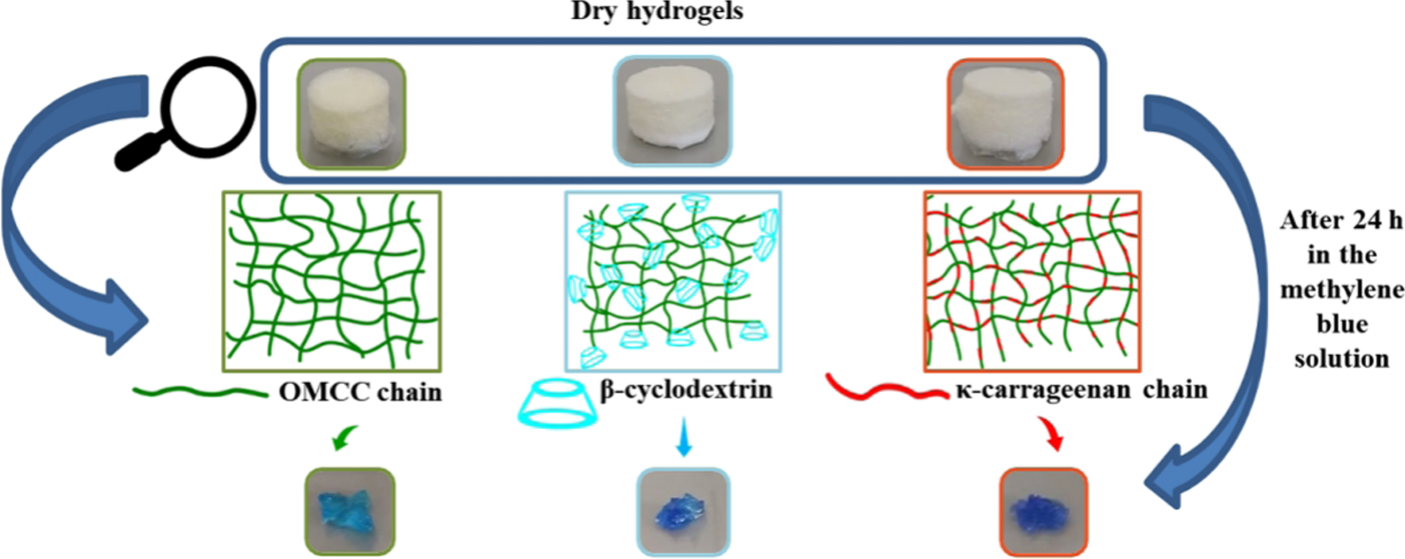

Cellulose-based materials are promising adsorbents for pollutants and other classes of compounds. Here, we report the preparation of hydrogels via chemical cross-linking of microcrystalline cellulose oxidized by the 2,2,6,6-tetramethylpiperidine-1-oxyl radical (TEMPO). The cross-linking process was carried out in the presence of modifiers such as β-cyclodextrin in order to insert hydrophobic cavities or κ-carrageenan due to the presence of negative charges along the molecular chains. Fourier transform infrared spectroscopy (FTIR) characterization evidenced the modification of cellulose chains. Swelling tests showed that all hydrogels reached the swelling equilibrium in the first 10 min of contact, while N_2_ adsorption isotherms allowed to evaluate surface areas ranging from 211 to 697 m^2^/g. Adsorption studies using methylene blue showed the key role of negative charges present in polymer chains favoring interactions with this cationic dye, as hydrogels modified with κ-carrageenan were found to have a high adsorption performance. The kinetic analysis of the adsorption process also suggests contributions from chemical interactions, which may involve electron transfer or electron pairing, as the data from all hydrogels showed high-quality fitting with the pseudo-second-order model.

## Introduction

Emerging pollutants such as heavy metals, dyes, pesticides, and pharmaceuticals have raised significant concern in recent years following the development of industrial and agricultural activities, which resulted in increasing pollution of water resources.^1,2^ Traditional approaches including chemical precipitation, membrane filtration, electrochemical treatment, and adsorption have been used to remove those pollutants, with adsorption being particularly advantageous due to the low cost and easy access.^3,4^ In this context, the choice of the adsorbent relies on criteria such as the affinity of active sites with the pollutant molecules. Active carbon, zeolite, polymer resins, silica gel, and diatomaceous earth are among the most important adsorbents, although chemical modifications are often necessary in order to improve the affinity for specific pollutants. Thus, the search for novel adsorbents combining facile preparation, high adsorption efficiency, reusability, and low environmental impact has become an intense research field.^5^ In this context, hydrogels are receiving increasing attention, since their structures can be rationally planned to ensure high adsorption capacity as well as highly porous structures containing functional suitable to capture the desired pollutant.^6^

Hydrogels based on renewable polymers are particularly fitted to match environmental demands, and among those polymers, cellulose is attractive owing to its abundance, biodegradability, low cost, presence in solid wastes, and amenability to chemical and physical transformations.^7^ Some modifications in the structure of native cellulose are necessary in order to separate the strongly packed cellulose chains and ensure hydrogel formation, including the oxidation of hydroxyl groups, yielding negatively charged carboxylates. Oxidation mediated by *N*-oxyl-2,2,6,6-tetramethylpiperidine (TEMPO) selectively modifies carbon 6 of cellulose into carboxyl or aldehyde groups. This process, in combination with mechanical treatment and centrifugation, enables the separation of cellulose fibrils into nanofibers, as reported in previous studies.^8^

TEMPO-oxidized cellulose is suitable to form hydrogels through physical and chemical cross-linking,^9−11^ with swelling and adsorption capacities that can be further improved upon insertion of specific functional groups. For instance, the insertion of molecular cavities able to accommodate hydrophobic molecules has been described through modification of TEMPO-oxidized cellulose with β-cyclodextrin,^12−14^ as it may provide additional sites of interaction with hydrophobic molecules.^15^ Regarding electrically charged species, hydrogel modification with oppositely charged groups may favor the adsorption process.^16,17^ For instance, the increase in negatively charged groups in oxidized cellulose hydrogels can also contribute to the adsorption performance toward cationic dyes. In this context, modification with carrageenan, a linear sulfated polysaccharide obtained from the red algae *Eucheuma spinosum* (*Eucheuma denticulatum*) or *Eucheuma cottonii* (*Kappaphycus alvarezii*),^18^ has been described as a strategy to improve the adsorption properties of several classes of hydrogels.^19^

Studies on hybrid hydrogels upon the combination of TEMPO-oxidized cellulose and nanocelluloses with other components including other polymers and graphene oxide are receiving attention in recent literature to adsorption of methylene blue, as poly(vinyl alcohol)^20^ and polyethylenimine-graphene oxide/cellulose nanofibril.^21^ Results show that the combinations of components are a successful strategy to improve the adsorption capacity. Here, we report the chemical cross-linking of TEMPO-oxidized microcrystalline cellulose unmodified and modified with β-cyclodextrin and κ-carrageenan. Effects of chemical modifications on textural properties, swelling degree, and thermal behavior were studied. Finally, the performance of different hydrogels on the adsorption of methylene blue and adsorption kinetics were also evaluated. The novelty of this study lies in the combined use of κ-carrageenan and β-cyclodextrin to chemically modify TEMPO-oxidized cellulose hydrogels with the aim of enhancing their adsorption properties. This work aims to develop hydrogels with improved structural and functional characteristics suitable for adsorption processes, offering an efficient alternative for the removal of cationic pollutants from aqueous solutions.

## Materials and Methods

### Materials

Microcrystalline cellulose (MCC, from cotton linters), *N*-oxyl-2,2,6,6-tetramethylpiperidine (TEMPO), β-cyclodextrin hydrate, and κ-carrageenan were purchased from Sigma-Aldrich. Epichlorohydrin (99%) was purchased from Synth. All of the other chemicals were used without further purification.

### TEMPO-Mediated Oxidation of Microcrystalline Cellulose

TEMPO-assisted oxidation of MCC was carried out according to the methodology proposed by Salminen et al.^22^ Briefly, an aqueous suspension of 5 g of MCC in 400 mL was treated in an ultrasonic bath (37 Hz) for 20 min. Then, 0.1 mmol of TEMPO per gram of cellulose and 1 g of NaBr per gram of cellulose were added to the MCC suspension. After stirring for 20 min, an 8% NaClO solution (15 mmol per gram cellulose) was added, and the pH was corrected to 10–11 with 1 mol L^–1^ NaOH. The pH was observed to have decreased in the course of the reaction and must be corrected until no further decrease, signalizing the end of the reaction. Then, the TEMPO-oxidized microcrystalline cellulose (TOMCC) was precipitated with the addition of 50 mL of ethanol, centrifuged, and washed with ethanol and distilled water 5 times. Finally, the TOMCC was frozen at −80 °C and freeze-dried. The degree of oxidation and carboxyl content were determined using the conductometric titration method.^23,24^

### Preparation of Hydrogels

TOMCC was cross-linked according to an adaptation of the method described by Zhang et al.^25^ Conditions are summarized in Table 1. The preparation of unmodified hydrogels was studied through the addition of 500 μL of epichlorohydrin to TOMCC suspensions at two different concentrations, namely, 6 and 9% in mass. For the modified hydrogels, half of the TOMCC mass was replaced by β-cyclodextrin or κ-carrageenan and added to a 7:12:81 (m/m) NaOH/urea/water solution precooled at −12 °C, followed by vigorous stirring for 5 min.^25^ Then, the cross-linker was added, and the mixture was stirred at room temperature for 20 min, transferred to a beaker, and heated at 60 °C for 1 h. Finally, the resulting hydrogels were immersed in 50 mL of distilled water, which was changed 5 times a day, and kept for 5 days to remove residual reagents. After the washing step, the hydrogels were freeze-dried prior to characterization. The hydrogels were named according to the amount of TOMCC used and the presence of β-cyclodextrin or κ-carrageenan, as shown in Table 1.

**Table 1:** Experimental Conditions Used for Cross-Linking TOMCC with Epichlorohydrin in the Presence of Modifiers

TOMCC (%)	β-cyclodextrin	κ-carrageenan	hydrogel
6	_	_	HOC6
6	50% cellulose mass	_	HOC6βCD
6	_	50% cellulose mass	HOC6κC
9	_	_	HOC9
9	50% cellulose mass	_	HOC9βCD
9	_	50% cellulose mass	HOC9κC

### Characterization

Samples were characterized by Fourier transform infrared spectroscopy (FTIR) spectroscopy in the form of KBr pellets using a PerkinElmer Spectrum Two in the 4000–400 cm^–1^ range, with 8 cm^–1^ resolution and 64 scans. Scanning electron microscopy (SEM) images for MCC and TOMCC were acquired in a JEOL microscope JSM 5700 applying a 10 kV voltage. Samples were deposited in stubs and metalized with silver. Transmission electron microscopy (TEM) images were obtained for TOMCC with a JEOL microscope JEM1400 Plus operating at 120 kV. Samples were suspended in deionized water, sonicated, and deposited onto copper grids coated with a thin layer of Formvar and carbon, followed by negative staining with 2% uranyl acetate. Images were analyzed with ImageJ software for measuring the fiber diameters. Solid-state ^13^C NMR data for MCC and TOMCC were acquired on a Burker AC-300 P NMR spectrometer using a 5 mm Bruker probe. Bloch decay was observed with high-power proton decoupling (HPDEC, with a pulse delay of 0.3 s); pulse sequences were used to acquire spectra at a spinning rate of 10 kHz. TG analyses were performed for MCC, TOMCC, and hydrogels with a NETZSCH instrument model STA 449 F1 Jupiter instrument applying a 10 °C/min heating rate under a N_2_ atmosphere. Nitrogen adsorption/desorption isotherms were obtained for hydrogels and determined at 77 K using a NOVA 1200e from Quantachrome Instruments. Samples were degassed under vacuum at 150 °C for 2 h before measurements, and data were treated with the Brunauer–Emmett–Teller (BET) equation to estimate surface area at relative pressures from 0.05 to 0.3. Pore size distribution was derived from adsorption branches using the Barrett–Joyner–Halenda (BJH) method. Diffraction patterns were obtained using a Rigaku X-ray diffractometer with Cu Kα radiation (λ = 1.5418 Å) at 40 kV voltage, 40 mA tube current, a 0.04° step size, and continuous scanning mode over a 2θ range of 5–45°.

### % Swelling

The water absorption capability of the hydrogels was determined by immersing approximately 100 mg of a given dry sample into 50 mL of distilled water, followed by measurements of the hydrogel mass at predetermined times. To accomplish the mass measurements, the hydrogel was withdrawn and the excess water was carefully wiped out with filtering paper before weighing in an analytical balance.^26,27^ The experiments were carried out in triplicate. The % swelling was calculated with eq 1
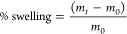
1where *m*_*t*_ is the hydrogel mass at time *t* and *m*_0_ is the mass of the dry sample.

### Adsorption Studies

Dry hydrogel samples were powdered using a portable mixer before adsorption, and the effects of pH and adsorbent mass were studied under 150 rpm stirring for 24 h at room temperature as follows. Solutions in which the MB concentration was fixed at 3 mg L^–1^^28^ were prepared using aqueous media with pH values (1.98, 3.74, 7.24, 8.23, and 9.95) previously adjusted with HCl or NaOH. The adsorbent mass was fixed at 10 mg for pH evaluation. The effect of different adsorbent masses (5, 10, and 20 mg) was evaluated using 3 mg L^–1^ MB solution at pH ≈ 7.0. After 24 h, the suspensions were centrifuged at 3900 rpm for 15 min, and the absorbance of the supernatant was read at 664 nm in a Rayleigh UV-9200 spectrophotometer. To evaluate the effect of initial MB concentrations (3 to 70 mg/L), 10 mg of the dry hydrogel was added to 10 mL of MB solution at pH ≈ 7.0. Following 1 h of agitation at 150 rpm, the samples were centrifuged at 3900 rpm for 5 min, and the supernatant absorbance was recorded at 664 nm using the same spectrophotometer.

The % removal of MB (%*R*) was calculated using eq 2
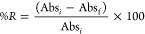
2where Abs_*i*_ is the initial absorbance of the MB solution and Abs_f_ is the absorbance after adsorption.

The removal efficiency (η(%)) was calculated using eq 3
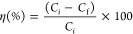
3where *C*_*i*_ and *C*_f_ are the initial and final concentrations (mg·L^–1^) of the MB solution.

### Adsorption Kinetics Study

10 mg of the dry hydrogel was added to 10 mL of MB solution at pH ≈ 7.0. After predetermined times, 1.5 mL aliquots were collected and centrifuged at 3900 rpm for 5 min, and the absorbance of the supernatant was read at 664 nm in a Rayleigh UV-9200 spectrophotometer. The adsorptive capacity *q*_*t*_ (mg·g^–1^) at time *t* (min) was calculated using eq 4
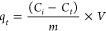
4where *C*_*i*_ and *C*_*t*_ are the MB concentrations (mg·L^–1^) at the beginning of the experiment (*t*_0_) and at subsequent times (*t*), *m* is the dry hydrogel mass (mg), and *V* is the volume of the solution (L). All experiments were carried out in triplicate.

Experimental data were evaluated kinetically using pseudo-first-order, pseudo-second-order, Elovich, and intraparticle diffusion models. The equations for these models are provided in the Supporting Information.

### Equilibrium Study

For the equilibrium study, experiments were conducted over 1 h, as determined in the kinetic analysis. The Langmuir and Freundlich adsorption isotherm models (with equations provided in the Supporting Information) were then applied to fit the process data using nonlinear regression analysis.

### Reusability Study

Five adsorption–regeneration cycles were performed in order to evaluate the reuse of hydrogels. In this study, 1 mg of the dry hydrogel was added to 10 mL of 3 mg L^–1^ aqueous MB solution. After 24 h of stirring at 150 rpm at room temperature, the hydrogels were removed and transferred to 20 mL of hydrochloric acid solution (0.2 mol·L^–1^) and left in contact for 24 h for regeneration. Then, the hydrogels were washed three times with distilled water and submitted to the next cycle. The absorbance at 664 nm was measured, and the removal efficiency was calculated using eq 3.

## Results and Discussion

**Figure 1:**
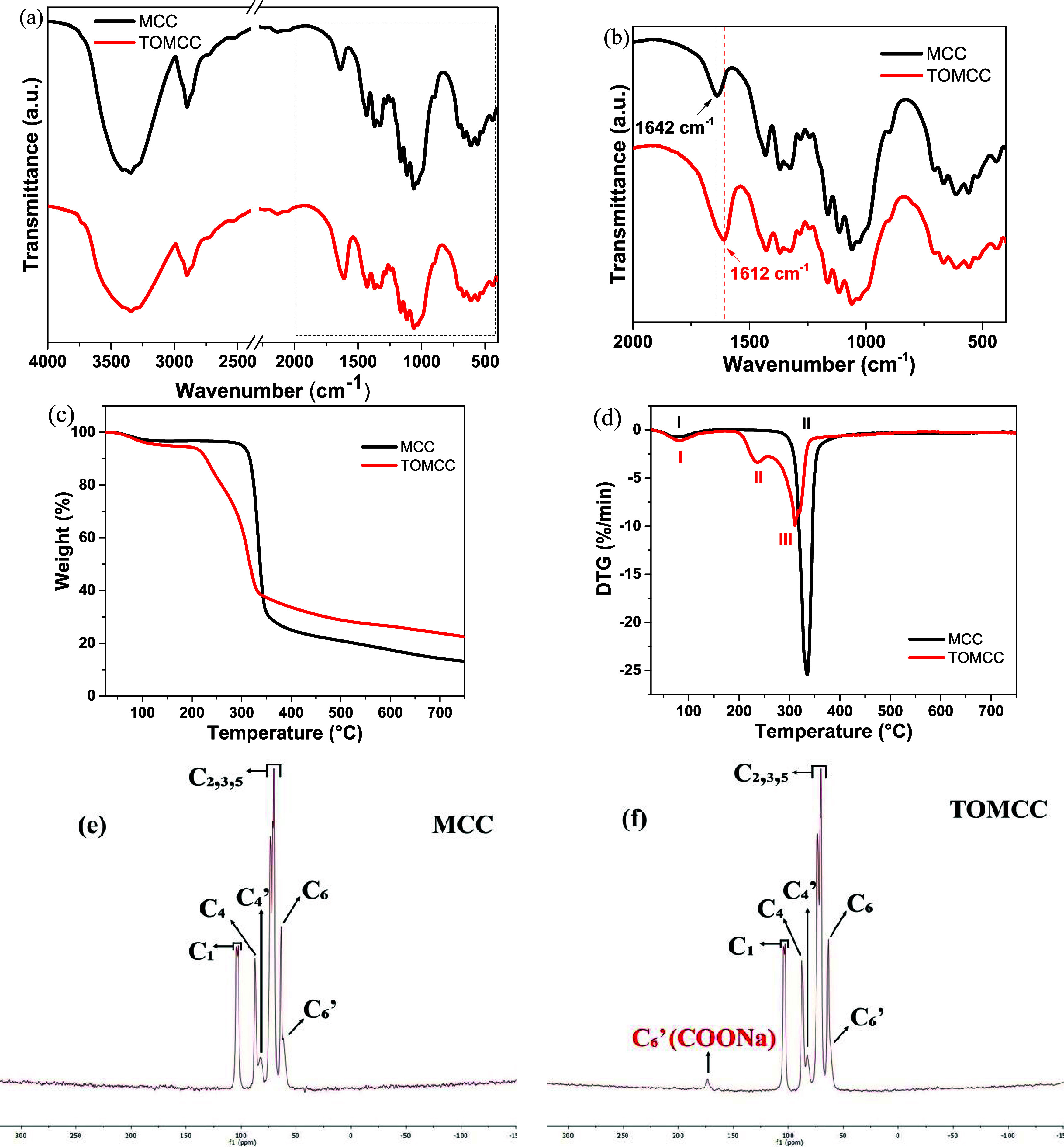
(a, b) FTIR spectra, (c) TG curves, (d) DTG curves for MCC and TOMCC, and (e, f) solid-state ^13^C NMR spectra of MCC and TOMCC.

FTIR spectroscopy, thermal analysis, and ^13^C NMR spectroscopy allowed the characterization of TOMCC samples. Pristine MCC structure bands at 3340 and 2900 cm^–1^ were assigned respectively to OH and CH stretching vibrations of the polysaccharide structure; a band from H–O–H bending at 1640 cm^–1^ evidenced the presence of adsorbed water^29−31^ in addition to bands related to CH_2_ deformation at the 1435–1327 cm^–1^ interval. Finally, additional bands can be observed at 1165 and 1070 cm^–1^ due to C–C and C–O (ether) bonds from the backbone, respectively. After oxidation with TEMPO, the main evidence of MCC oxidation was the replacement of the band at 1642 cm^–1^ by the band at 1612 cm^–1^, Figures 1a,b, which is assigned to the asymmetric stretching of the carboxylate COO– group.^32−35^ Those groups are present, since the oxidation occurs under an alkaline medium and the carboxylic acid groups are converted into their salt forms. As the band at 1612 cm^–1^ has a higher intensity than that at 1642 cm^–1^, the latter is masked after oxidation. All those observations confirm the success of the oxidation, in agreement with the literature.^36−38^ The other spectral regions remained mainly unchanged. The carboxylate content and degree of oxidation for MCC were 0.10 mmol·g^–1^ and 0.02, respectively. For TOMCC, these values increased to 0.69 mmol·g^–1^ and 0.11, further confirming, alongside FTIR analysis, the presence of sodium carboxylate groups in cellulose after oxidation.

When analyzing the thermogravimetric curves (Figure 1c), the first degradation event observed for both MCC and TOMCC is the release of water molecules below 150 °C. Additionally, MCC has the highest mass loss (72.3%) from 275 to 413 °C related to cellulose pyrolysis, with a maximum degradation temperature (*T*_max_) of 335 °C. With the oxidation of microcrystalline cellulose, the *T*_max_ is reduced to 310 °C with a maximum weight loss of 43.2%. Furthermore, a new event is observed only for TOMCC below from 188 to 257 °C, with a mass loss of 14.6%. This event may be associated with decarboxylation reactions of the anhydroglucose units on the surface of TOMCC.^33,39^

Solid-state ^13^C NMR spectra (Figure 1e,f) for MCC and TOMCC exhibit typical peaks of cellulose units in the range of 110–65 ppm. Signals assigned to C1 are observed from 102 to 104 ppm, while those from C2, C3, and C5 carbons occur in the range of 70–73 ppm.^40,41^ As MCC presents both crystalline and amorphous regions, the signals assigned to C4 from crystalline and amorphous regions (labeled as C4′) are observed at around 87 and 82 ppm, respectively. Analogously, the C6 and C6′ resonances appear at 63 and 61 ppm, respectively.^42^ After MCC oxidation, an additional signal appears at 173 ppm, which is attributed to the carbon atom in the sodium carboxylate groups. Furthermore, the intensity of the peak at 61 ppm reduced after oxidation, suggesting preferential oxidation of C6 hydroxyl groups in amorphous regions.^43,44^

**Figure 2:**
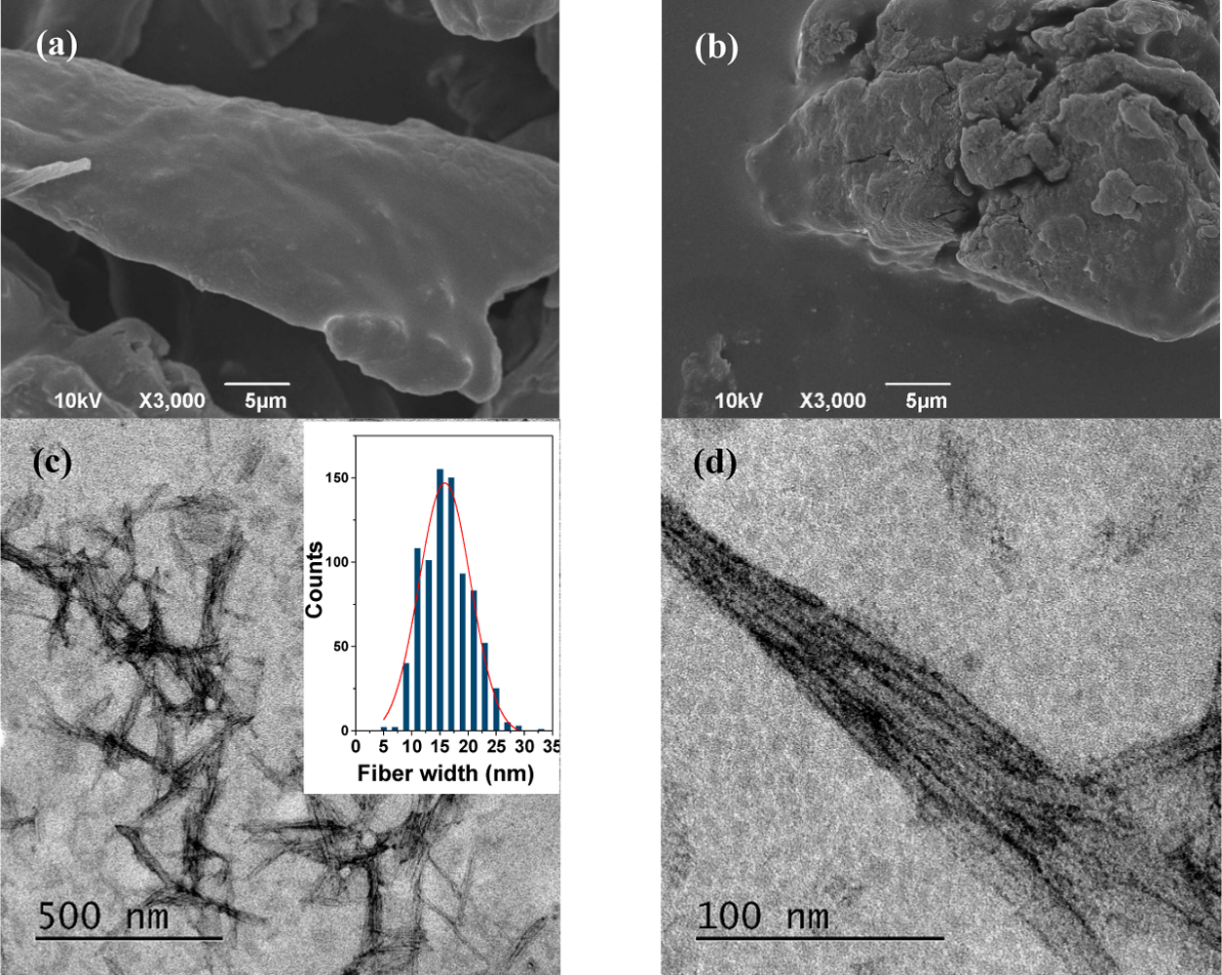
(a) SEM image of MCC. (b) SEM image of TOMCC. (c) TEM images for TOMCC (inset: histogram showing fiber diameter distribution). (d) TEM image of TOMCC.

The typical morphology of MCC under SEM observation (Figure 2a) shows particles with irregular shapes and slightly rough surfaces.^45,46^ After oxidation (Figure 2b), the roughness of the surface increases, and the particles are fragmented with the presence of voids and cracks. According to Rossetti et al.,^47^ the introduction of carboxylate groups in cellulose increases the negative charges on the surface, causing repulsion between the fibers. The process also leads to the separation of cellulose nanofibers, which can be observed in TEM images (Figure 2c,d) using negative staining with uranyl acetate. TEM images show nanofibers with an average width of 15.89 ± 0.31 nm and variable lengths, which form bundles of primary fibrils.

**Figure 3:**
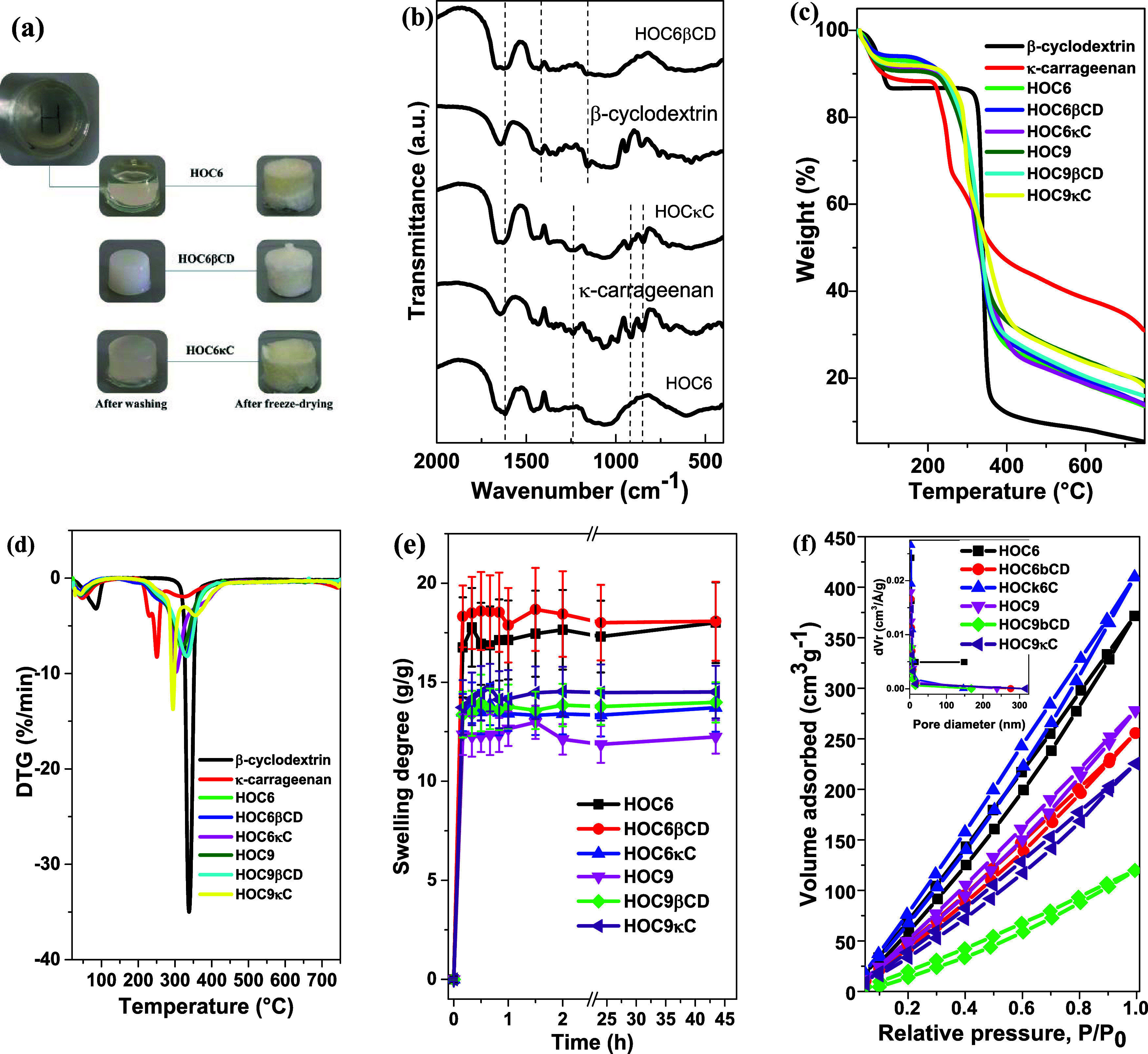
(a) Photographs of hydrogels during the washing process and after freeze-drying obtained with 6% MOCC and modified with κ-carrageenan and β-cyclodextrin. (b) FTIR spectra hydrogels obtained with 6% MOCC and modified with κ-carrageenan and β-cyclodextrin. (c) TG curves and (d) DTG curves for hydrogels obtained with 6 and 9% MOCC before and after modification with β-cyclodextrin and κ-carrageenan. (e) Swelling degree curves for hydrogels obtained with 6 and 9% MOCC before and after modification with β-cyclodextrin and κ-carrageenan. (f) Nitrogen adsorption/desorption isotherms for hydrogels obtained with 6% and 9% MOCC before and after modification with β-cyclodextrin and κ-carrageenan (inset shows the pore size distribution graph).

Monolithic hydrogel samples (Figure 3a) were obtained after cross-linking with epichlorohydrin in the absence and presence of modifiers. Samples were characterized in order to identify the functional groups present as well as thermal, textural, and swelling properties. FTIR spectroscopy was insensitive to differences in TOMCC amount; thus, only data for samples with 6% TOMCC are shown (Figure 3b). Unmodified hydrogels exhibited spectra essentially identical to that of TOMCC. Spectra from both κ-carrageenan and β-cyclodextrin are characterized by bands from the carbohydrate structure, with differences at 400–1500 cm^–1^ for β-cyclodextrin (combined vibrational modes including C–O stretching and C–O–H bending) and at 1220, 928, and 844 cm^–1^ for κ-carrageenan (all assigned to modes involving ester sulfate groups).

TG (Figure 3c) and DTG (Figure 3d) allow a comparison of the thermal behavior of modified hydrogels with the original modifiers, showing that the thermal behavior of hydrogels is distinct compared to the sole modifiers. TG curves show that the thermal behavior of hydrogels with β-cyclodextrin is similar to hydrogels containing only TOMCC, except for a slight increase in thermal stability. The main degradation event with a weight loss of ≈65% occurred in the temperature range of 150–445 °C with *T*_max_ ≈ 331 °C, while the hydrogels-only TOMCC was *T*_max_ ≈ 325 °C. In contrast, hydrogels with κ-carrageenan exhibited a reduction in thermal stability with the main degradation event at *T*_max_ ≈ 303 °C for HOC6κC and *T*_max_ ≈ 230 °C for HOC9κC.

Figure 3e shows that all hydrogels reach swelling equilibrium during the first 10 min of contact. For unmodified hydrogels, increasing the amount of TOMCC decreased the swelling degree. Similar behavior was observed by Chang et al.^48^ and Butrim et al.,^49^ resulting from the increase of hydrogen bonding sites as well as entanglement of cellulosic chains. Both processes are favored by the shorter distances between the cellulose fibers that occur at higher concentrations.^48^ The same trend was found for β-cyclodextrin-modified hydrogels, suggesting that hydroxyl groups from β-cyclodextrin contribute to intermolecular interactions via hydrogen bonding in an analogous fashion as TOMCC. On the other hand, the swelling was independent of the TOMCC amount for κ-carrageenan-modified hydrogels, suggesting that the repulsive role of negative charges present in κ-carrageenan overcomes that of hydrogen bonding.

N_2_ adsorption/desorption isotherms were essentially identical for all hydrogels (Figure 3f), and although the isotherms did not fit into any model types described by IUPAC, valuable information is provided. Samples start adsorbing from very low relative pressures, evidencing the crucial role of interactions of the gas with the surface of the solids. Also, this observation suggests the presence of mesopores, although no clear separation between monolayer and multilayer stages can be seen. Adsorption progressively increases at intermediate relative pressure values, which also evidence the presence of mesopores and suggests the formation of a multilayer, progressing without a final plateau, indicating that the external surface is available for adsorption. Thus, the isotherm profiles are consistent with a broad pore size distribution (Figure 3f), and the calculation of textural parameters using the Barrett–Joyner–Halenda (BJH) equation indicates predominant mesoporosity (Table 2).

**Table 2:** Textural Data for Hydrogels

sample	surface area (m^2^/g)	pore volume (cm^3^/g)	pore diameter (nm)
HOC6	622	0.509	3.634
HOC6βCD	440	0.358	3.608
HOC6κC	698	0.565	3.621
HOC9	484	0.385	3.631
HOC9βCD	211	0.172	3.617
HOC9κC	383	0.318	3.630

The materials have surface areas (Table 2) above 200 m^2^·g^–1^, which can be considered large for this kind of material^50^ and may be attributed to the presence of cellulose nanocrystals after oxidation.^10,50^ Here, we found that an increase in the concentration of TOMCC reduced the surface area of the hydrogels. Possibly, the lower distances between the fibers favored agglomeration during the cross-linking process, reducing the void spaces and consequently the surface area. The reduction of the surface area with the increase in the concentration of cellulose was also observed by Karadagli et al.,^51^ reporting 206 m^2^·g^–1^ for the lowest concentration of cellulose and 159 m^2^·g^–1^ for the sample with the highest concentration. Finally, the effect of modifiers depended on the TOMCC amount: it further decreased the surface area for 9% TOMCC, while for 6% TOMCC, the effect reflected the type of modifier. Addition of β-cyclodextrin decreased the surface area, possibly as a result of agglomeration, while addition of κ-carrageenan had the opposite effect, probably due to interchain electrostatic repulsion.

The diffractograms (Supporting Information) of hydrogels composed solely of TOMCC displayed broad peaks at 2θ ≈ 12.3 and 20.2°, characteristic of the cellulose II structure.^52,53^ After the incorporation of β-cyclodextrin and κ-carrageenan, a shift in the 20.2° peak to 20.9° was observed, along with the appearance of a new peak at 2θ ≈ 23.1°. For the hydrogels containing β-cyclodextrin, the peak at 20.9° exhibited higher intensity compared to those with κ-carrageenan.

### Methylene Blue Adsorption Study

**Figure 4:**
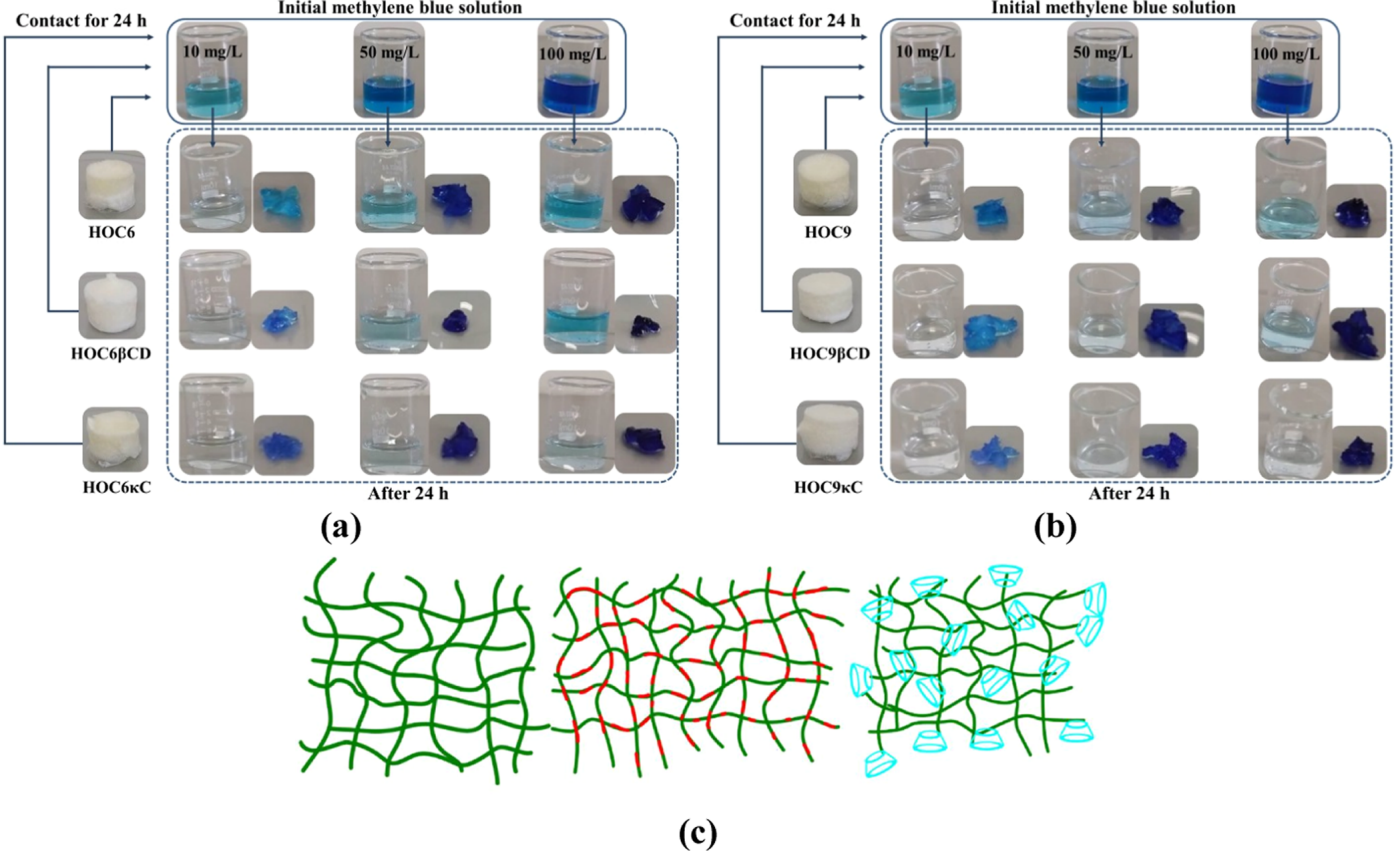
(a, b) Photographs of hydrogels before and after adsorption of methylene blue; (c) representation of unmodified and modified hydrogels where the representations stand for 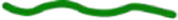: TOMCC chain, 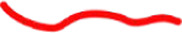: κ-carrageenan chain; 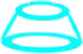: β-cyclodextrin.

All hydrogels prepared here were evaluated in the adsorption of a cationic dye, methylene blue (MB), chosen due to its high water solubility, ease of quantification, and well-established behavior in adsorption studies. Images of representative samples and solutions before and after contact with the hydrogels are shown in Figure 4a,b. Visually, all hydrogels can adsorb MB, although after 24 h, the colors of unmodified hydrogels were lighter, while with κ-carrageenan-modified, they acquired the most intense colors. Pictorial representations of unmodified and modified hydrogels are shown in Figure 4c.

**Figure 5:**
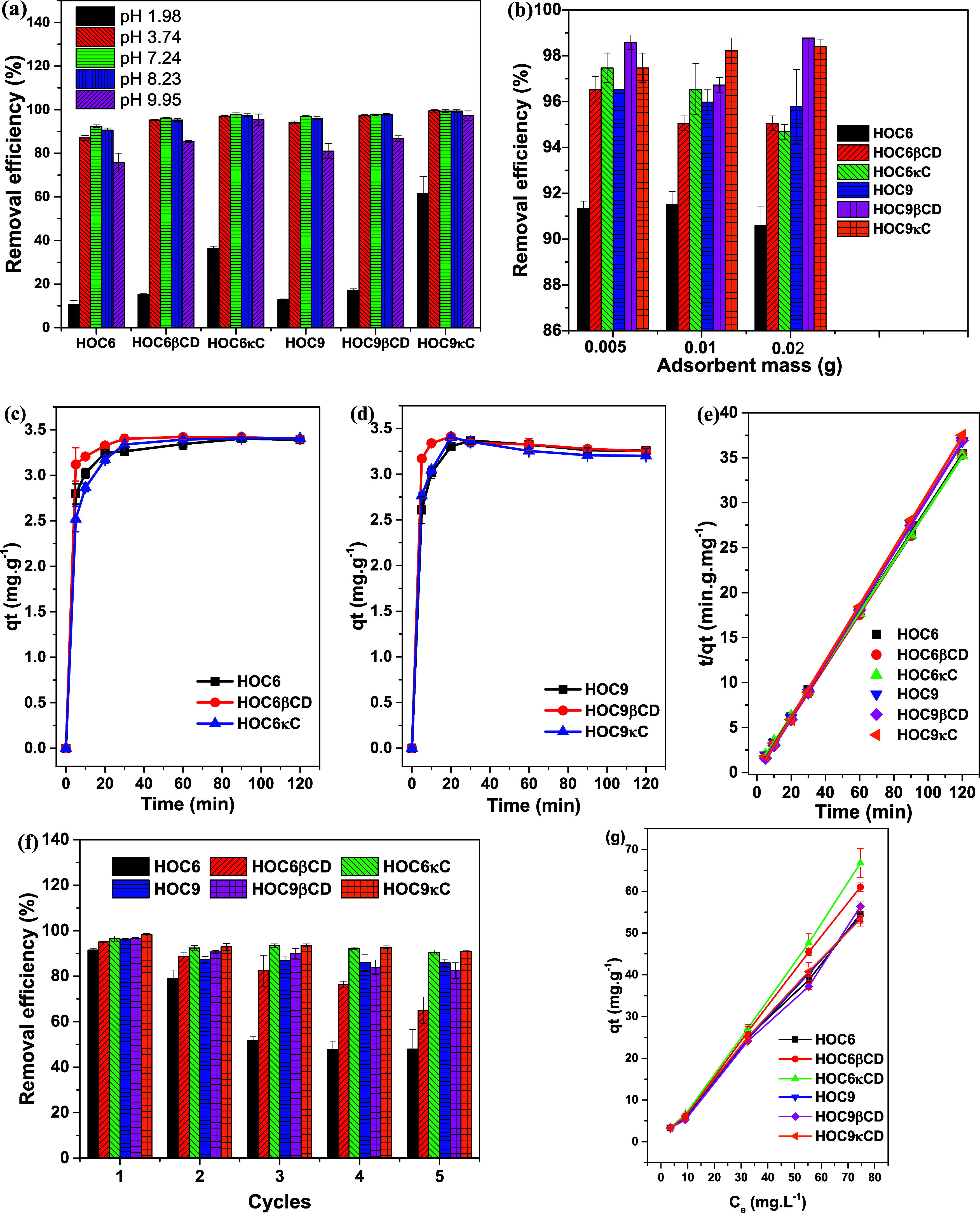
(a) Effect of pH on the removal efficiency for different adsorbents, (b) effect of adsorbent mass on the removal efficiency for different adsorbents, (c, d) experimental plots showing adsorbed amount as a function of time for different adsorbents, (e) linear plots of pseudo-second-order kinetics for different adsorbents, (f) efficiency removal of MB by hydrogels during regeneration cycles, and (g) effect of MB initial concentration on the MB adsorption capacity of the hydrogels.

The effects of the solution pH (Figure 5a) and adsorbent mass (Figure 5b) were evaluated. Regarding the pH effect, all hydrogels had the worst performances at pH 1.98. The low removal in a more acidic medium may be related to the unavailability of active sites, since the hydronium H_3_O^+^ ions and cationic dye molecules compete for the carboxylate and hydroxyl groups on the surface of TOMCC, in addition to OH groups in β-cyclodextrin and sulfate groups in κ-carrageenan.^54^ At pH 9.95, removal efficiency slightly decreased for both unmodified hydrogels and those modified with β-cyclodextrin, which may be associated with increased repulsive forces due to high OH^–^ concentration.^55,56^ In the pH range between 3.74 and 8.23, all hydrogels removed more than 80% of the dye. The hydrogels modified with κ-carrageenan were less sensitive to pH, even under a strongly acidic medium. Considering that all hydrogels had a satisfactory performance at pH 7.24, this value was chosen for further investigation. The effect of the adsorbent mass depended on the nature of the hydrogel. Unmodified hydrogels showed very similar removal efficiencies independent of the adsorbent mass with the improvement of the efficiency for the higher TOMCC amount. Regarding modified hydrogels, the general trend pointed to both the role of TOMCC percentage and the presence of modifiers in increasing removal efficiency.

Several factors, including the surface area, swelling degree, and chemical nature of the surface at each pH value, can affect the removal efficiency. The hydrogel with the best performance was HOC9κC, which has neither the highest surface area nor the highest swelling degree, suggesting the effect of the chemical nature. The p*K*_a_ values of sulfate and carboxylic groups are 2.8 and 3.6, respectively,^57^ which may explain the better removal efficiency of the hydrogels modified with κ-carrageenan at lower pH. Possibly, it may be partially deprotonated and negatively charged, favoring interaction with the cationic dye. The other hydrogels have carboxylate groups, and those modified with β-cyclodextrin can accommodate dye molecules in the supramolecular cavities. In summary, the decreasing order of efficiency observed here is the electrostatic interaction of positively charged MB with negative sulfate and carboxylic groups > electrostatic interaction of positively charged MB with negative carboxylic groups plus hydrophobic interactions with β-cyclodextrin cavities > electrostatic interaction of positively charged MB with negative carboxylic groups in unmodified hydrogels.

The adsorption capacity of the hydrogels over time (Figure 5c,d) was evaluated. After 20 min, all hydrogels approached adsorption equilibrium, indicating a rapid adsorption process. Adsorbed amount data as a function of time were used to test pseudo-first-order, pseudo-second-order, Elovich, and intraparticle diffusion kinetic models, applying nonlinear and linear equations. Figure 5e shows experimental data and linear pseudo-second-order plots, which exhibited the best agreement with experimental data. Other graphs are available in the Supporting Information. Experimental data show that hydrogels modified with β-cyclodextrin have higher removal efficiencies at short times, suggesting that interaction with the cavities occurs at higher rates, although all hydrogels with 6% TOMCC reached equilibrium after 80 min, while those with 9% TOMCC reached after 30 min. The use of the kinetic model expressions yielded values presented in Table 3 for a nonlinear fit and in Table 4 for a linear fit. In general, the experimental data fitted well to pseudo-first-order, pseudo-second-order, and Elovich kinetic models, especially when evaluated by nonlinear regression. However, they indicated a slight preference for the pseudo-second-order model. It is worth noting that nonlinear models are generally more robust and precise than linear models, as they better represent the nature of the experimental data.

**Table 3:** Kinetic Parameters from Nonlinear Regression

		hydrogel					
model	parameter	HOC6	HOC6βCD	HOC6κC	HOC9	HOC9βCD	HOC9κC
_	*q*_e_ exp (mg·g^–1^)	3.390	3.395	3.406	3.256	3.251	3.201
pseudo-first order	*q*_e_ (mg·g^–1^)	3.306	3.370	3.329	3.291	3.326	3.266
	*k*_1_ (min^–1^)	0.350	0.503	0.254	0.301	0.615	0.359
	RSS	0.060	0.029	0.090	0.025	0.016	0.054
	*R*^2^	0.993	0.997	0.990	0.997	0.998	0.994
pseudo-second order	*q*_e_ (mg·g^–1^)	3.417	3.432	3.492	3.398	3.336	3.338
	*k*_2_ (g·mg^–1^·min^–1^)	0.252	0.538	0.145	0.219	1.752	0.332
	RSS	0.004	0.005	0.008	0.051	0.027	0.084
	*R*^2^	0.999	0.999	0.999	0.994	0.997	0.991
Elovich	α	2.9 × 10^5^	8.6 × 10^12^	9.7 × 10^2^	3.5 × 10^5^	3.3 × 10^43^	8.8 × 10^9^
	β	5.510	10.680	3.660	5.656	32.421	8.967
	RSS	0.029	0.014	0.093	0.179	0.040	0.176
	*R*^2^	0.997	0.999	0.990	0.980	0.996	0.981
intraparticle diffusion	*k*_d_ (mg/g-^1^·min^0.5^)	0.127	0.204	0.232	0.209	0.182	0.195
	C	1.628	1.801	1.498	1.631	1.898	1.717
	*R*^2^	0.495	0.218	0.592	0.466	0.340	0.410

**Table 4:** Kinetic Parameters from Linear Regression

			hydrogel					
model	parameter		HOC6	HOC6βCD	HOC6κC	HOC9	HOC9βCD	HOC9κC
	*q*_e_ exp (mg·g^–1^)		3.390	3.395	3.406	3.256	3.251	3.201
pseudo-first order	*q*_e_ (mg·g^–1^)		1.004	1.527	1.604	2.935	3.251	2.731
	*k*_1_ (min^–1^)		0.059	0.175	0.087	0.261	0.739	0.303
	RSS		2.637	1.602	1.185	0.065	0.000	0.151
	*R*^2^		0.705	0.808	0.939	0.981	1.000	0.968
pseudo-second order	*q*_e_ (mg·g^–1^)		3.432	3.421	3.465	3.279	3.251	3.205
	*k*_2_ (g/(mg·min^–1^))		0.226	0.746	0.171	1.259	–0.545	–1.089
	RSS		0.045	0.066	0.072	0.391	0.164	0.424
	*R*^2^		1.000	0.999	0.999	0.999	0.999	0.999
Elovich	α		2.9 × 10^5^	8.6 × 10^12^	9.8 × 10^2^	3.5 × 10^5^	6.4 × 10^164^	8.8 × 10^9^
	β		5.510	10.680	3.660	5.656	117.371	8.967
	RSS		0.029	0.014	0.093	0.179	0.0358	0.176
	*R*^2^		0.903	0.840	0.868	0.588	0.0164	0.366
intraparticle diffusion	stage I	*k*_d_ (mg/g^–1^·min^0.5^)	1.010	1.085	0.947	0.993	1.123	1.014
		*C*	0.122	0.157	0.091	0.088	0.150	0.113
		*R*^2^	0.952	0.931	0.969	0.973	0.942	0.959
	stage II	*k*_d_ (mg/g^–1^·min^0.5^)	0.107	0.084	0.206	0.156	0.006	0.141
		*C*	2.710	2.943	2.226	2.547	3.341	2.654
		*R*^2^	0.849	0.996	0.991	0.929	0.025	0.071
	stage III	*k*_d_ (mg/g^–1^·min^0.5^)	0.025	–0.001	0.012	–0.022	–0.018	–0.029
		*C*	3.139	3.415	3.283	3.490	3.450	3.499
		*R*^2^	0.872	0.009	0.833	0.955	0.957	0.917

When the intraparticle diffusion model was evaluated using nonlinear fitting, the results did not show a good fit. To enable a more comprehensive analysis of the characteristics and dynamics of the adsorption process, linear fitting was applied, revealing three distinct adsorption stages for all of the hydrogels. In Stage I, intraparticle diffusion was not predominant, as the C values were different from zero. However, adsorption occurred more rapidly compared to Stage II, where the diffusion coefficients decreased, suggesting a reduction in diffusion rates until equilibrium was reached. In Stage III, negative kid values were observed, suggesting that intraparticle diffusion is not the main rate-controlling step in the adsorption system analyzed.

Tables 3 and 4 show that *R*^2^ values from linear and nonlinear pseudo-second-order models were found to be very similar to each other. In this context, Lin and Wang performed a detailed comparison between linear and nonlinear pseudo-first- and pseudo-second-order kinetic models to fit MB adsorption onto activated carbon and observed that the difference in *q*_e_ (equilibrium adsorbed amount) between experimental and theoretical values was a better parameter than *R*^2^ to determine the best-fitting equation.^58^ Based on this, the authors concluded that the best-fitting nonlinear forms were superior to the linear forms. The model parameters might be distorted when the nonlinear equations were changed to yield the linear forms. The authors also stated that the nonlinear kinetic equations had the advantage of avoiding the need to know the value of *q*_e,exp_ before fitting the experimental points. Thus, based on all those considerations, we suggest that the nonlinear PSO model is more appropriate to determine the best-fitting equation. Thus, this indicates that the rate-determining step can be proposed as a chemical reaction between the adsorbent and the adsorbate, i.e., chemisorption.^59^ However, some authors recall that in light of the range of possible contributions to the kinetic behavior, the sole fit to a specific model does not evidence all fundamental aspects of the process.^60^ In practice, a better fit to pseudo-second-order than to pseudo-first-order indicates that the rate of uptake decelerates with time to a higher degree than would be expected in pseudo-first-order, which can also include contributions of slow diffusion within a network of fine pores.

The linear behavior observed between the adsorbent and different initial concentrations of MB (Figure 5g) demonstrates that the amount of the adsorbate retained per unit mass of the adsorbent is proportional to the equilibrium concentration of the adsorbate in the liquid phase. This experimental linearity suggests that within the concentration range tested, the system did not reach significant saturation. By applying the Langmuir and Freundlich isotherm models (plots in the Supporting Information) and analyzing the correlation coefficients (*R*^2^) shown in Table 5, it was found that the Freundlich model provided a better fit to the experimental data. This indicates that the system exhibits a heterogeneous adsorption surface with multilayer adsorption.^61^ This heterogeneity may suggest that adsorption occurs on sites of varying affinities, allowing the system to accommodate different dye concentrations without quickly reaching saturation.

**Table 5:** Langmuir and Freundlich Isotherm Model Parameters from Nonlinear Regression for MB Adsorption onto the Prepared Hydrogels

	Langmuir			Freundlich		
hydrogel	*R*^2^	*q*_max_ (mg·g^–1^)	*K*_L_ (L·mg^–1^)	*R*^2^	*n*_F_	*K*_F_ ((mg·g^–1^)·(L·mg^–1^)^1/*n*^)
HOC6	0.973	190.850	0.005	0.997	1.012	0.762
HOC6βCD	0.974	380.708	0.002	0.999	0.974	0.733
HOC6 κC	0.858	170.587	0.006	0.999	0.922	0.618
HOC9	0.996	221.444	0.004	0.998	1.018	0.780
HOC9βCD	0.969	816.048	0.001	0.993	0.928	0.529
HOC9κC	0.905	438.985	0.002	0.999	1.058	0.911

### Reusability Study

Studies on the reuse of adsorbents are a mandatory evaluation when considering the applicability of an adsorbent.^62^ Thus, we here evaluated the reuse of unmodified and modified hydrogels, using 0.2 mol·L^–1^ HCl solution as a hydrogel regeneration agent (Figure 5f). After five cycles of reuse, most hydrogels preserved methylene blue removal efficiency higher than 65% (except for the HOC6 hydrogel). The indicates that the hydrogels that kept high efficiency have potential for possible industrial application as high-performance recyclable adsorbents.

Table 6 brings a comparison of the performance of hydrogels reported here with literature reports on adsorbents based on cellulose, β-cyclodextrin, and κ-carrageenan, evidencing that the hydrogels prepared in this work can be considered high-quality and economically advantageous adsorbents. The materials were rationally designed to exhibit favorable adsorption properties and the approach proved successful, as observed by the fast adsorption process as well as the competitive adsorption efficiency.

**Table 6:** Comparison Study of MB Adsorption

adsorbent	removal efficiency (%)	refs
HOC6	91.5	this work
HOC6βCD	95.0	
HOC6κC	96.5	
HOC9	95.9	
HOC9βCD	96.7	
HOC9κC	98.2	
carboxymethylcellulose and sugar cane bagasse	90–95	(63)
gelatin, chitosan, and β-cyclodextrin	95.5	(16)
silica and β-cyclodextrin	95	(64)
cellulose and β-cyclodextrin	97	(65)
carboxymethylcellulose, κ-carrageenan, and montmorillonite clay	98	(19)
chitosan, κ-carrageenan, and bentonite	98.5	(66)

## Conclusions

The preparation of unmodified and modified hydrogels was achieved upon cross-linking with epichlorohydrin, and the presence of β-cyclodextrin and κ-carrageenan in TOMCC-based hydrogels was confirmed by FTIR spectroscopy. The presence of those modifiers altered characteristics such as thermal behavior, swelling degree, and surface area owing to the influence of attractive (β-cyclodextrin) and repulsive (κ-carrageenan) interchain interactions. Adsorption studies revealed that, in general, all hydrogels showed removal efficiency above 90%. Also, it is possible to conclude that increasing the concentration of TOMCC and the addition of modifiers improved the removal efficiency for methylene blue, with better results being found for κ-carrageenan, which confirms our hypothesis and suggests an important role for electrostatic interactions. The kinetic analysis of the adsorption process also suggests contributions of chemical interaction in the adsorption of methylene blue, since the data from all hydrogels show a high-quality fit with the pseudo-second-order model.

## Supporting Information Available

Equations used in the adsorption study, along with plots showing the application of kinetic models; isotherm graphs; and XRD patterns obtained for different hydrogels (PDF)

## Notes

**Consent for publication** All authors read, revised, and approved the final manuscript.

## Abbreviations

**MCC**: microcrystalline cellulose
**TOMCC**: TEMPO-oxidized microcrystalline cellulose
**TEMPO**: 2,2,6,6-tetramethylpiperidine-1-oxyl radical
**NaBr**: sodium bromide
**NaClO**: sodium hypochlorite
**NaOH**: sodium hydroxide
**MB**: methylene blue
**HCl**: hydrochloric acid
**HOC6**: hydrogel with 6% oxidized microcrystalline cellulose
**HOC6βCD**: hydrogel with 6% oxidized microcrystalline cellulose and the addition of β-cyclodextrin
**HOC6κC**: hydrogel with 6% oxidized microcrystalline cellulose and the addition of κ-carrageenan
**HOC9**: hydrogel with 9% oxidized microcrystalline cellulose
**HOC9βCD**: hydrogel with 9% oxidized microcrystalline cellulose and the addition of β-cyclodextrin
**HOC9κC**: hydrogel with 9% oxidized microcrystalline cellulose and the addition of κ-carrageenan
**FTIR**: Fourier transform infrared
**TEM**: transmission electron microscopy
**SEM**: scanning electron microscopy
^**13**^**C NMR**: carbon-13 nuclear magnetic resonance
**TG**: thermogravimetric
**BET**: Brunauer–Emmett–Teller
**BJH**: Barrett–Joyner–Halenda
**IUPAC**: International Union of Pure and Applied Chemistry
