# Supplementary material for: Preparation of TEMPO-Oxidized Cellulose Hydrogels Modified with β-Cyclodextrin and κ-Carrageenan for Potential Adsorption Applications
Source: ACS Omega. 2024 Dec 28;10(1):972–84. doi: 10.1021/acsomega.4c08188 (PMC11740384; doi:10.1021/acsomega.4c08188)
Supplement: Supplementary file 1 — ao4c08188_si_001.pdf [file ao4c08188_si_001.pdf]

Supplementary Information

ACS Omega

Preparation of TEMPO-Oxidized Cellulose Hydrogels Modified with β-Cyclodextrin and κ-Carrageenan for Potential Adsorption Applications

Liliane Oliveira Mota

Department of Materials Science and Engineering, Federal University of Sergipe, 49400-000, São Cristóvão – SE, Brazil

l.o.mota@hotmail.com

Yslaine Andrade de Almeida

Department of chemistry, Federal University of Sergipe, 49400-000, São Cristóvão – SE, Brazil

Diego Fonseca Bispo

Department of chemistry, Federal University of Sergipe, 49400-000, São Cristóvão – SE, Brazil

Marcos Fabio Farias Souza

Department of Chemical Engineering, Federal University of Sergipe, 49400-000, São Cristóvão – SE, Brazil

Douglas Costa Santos

Department of Chemical Engineering, Federal University of Sergipe, 49400-000, São Cristóvão – SE, Brazil

Raimundo Alves Lima Sobrinho

Department of Chemical Engineering, State University of Santa Cruz, 45662-900, Ilhéus – BA, Brazil

Iara F. Gimenez*

Department of chemistry, Federal University of Sergipe, 49400-000, São Cristóvão – SE, Brazil
iara.gimenez@gmail.com

Experimental data of adsorption kinetics were evaluated using pseudo-first-order, pseudo-second-order, Elovich and intraparticle diffusion models. Lagergren's pseudo-first-order

model can be expressed in its nonlinear form by the expression (S1) or linearly by equation (S2):

$$q_t = q_e(1-e^{-k_1 t}) \tag{S1}$$

$$\ln\left(q_e - q_t\right) = \ln(q_e) - k_1 t \tag{S2}$$

where k_1 is the first-order rate constant (min^{-1}), q_e and q_t are the adsorbed amounts (mg·g^{-1}) at equilibrium and at the time t, respectively. Similarly, the pseudo-second-order model can also be expressed by a nonlinear equation (S3) or linearly by the equation (S4):

$$q_t = \frac{q_e^{\,2} k_2 t}{1 + q_e k_2 t} \tag{S3}$$

$$\frac{1}{q_t} = \frac{1}{k_2 q_e^{\,2}} + \frac{t}{q_e} \tag{S4}$$

where k_2 is the adsorption second order rate constant (g·mg^{-1}·min^{-1}).

The Elovich model can be used in a nonlinear from according to equation (S5):

$$q_t = \frac{1}{\beta}\ln\left(1+\alpha\beta t\right) \tag{S5}$$

and also in a linear from as equation (S6) considering $t >> 1/\alpha\beta$ (27):

$$q_t = \frac{1}{\beta}\ln\left(\alpha\beta\right) + \frac{1}{\beta}\ln(t) \tag{S6}$$

where α is the initial adsorption rate (mg·g^{-1}·min^{-1}) and β is the desorption constant (mg·g^{-1}).

The intraparticle diffusion model described by Weber and Morris (1963) from according to equation (S7):

$$q_t = k_d t^{0.5} + C \tag{S7}$$

where k_d is intraparticle diffusion coefficient (mg·g^{-1}·min$^{-0.5}$) and C constant measures the thickness of the layer (mg·g^{-1}).

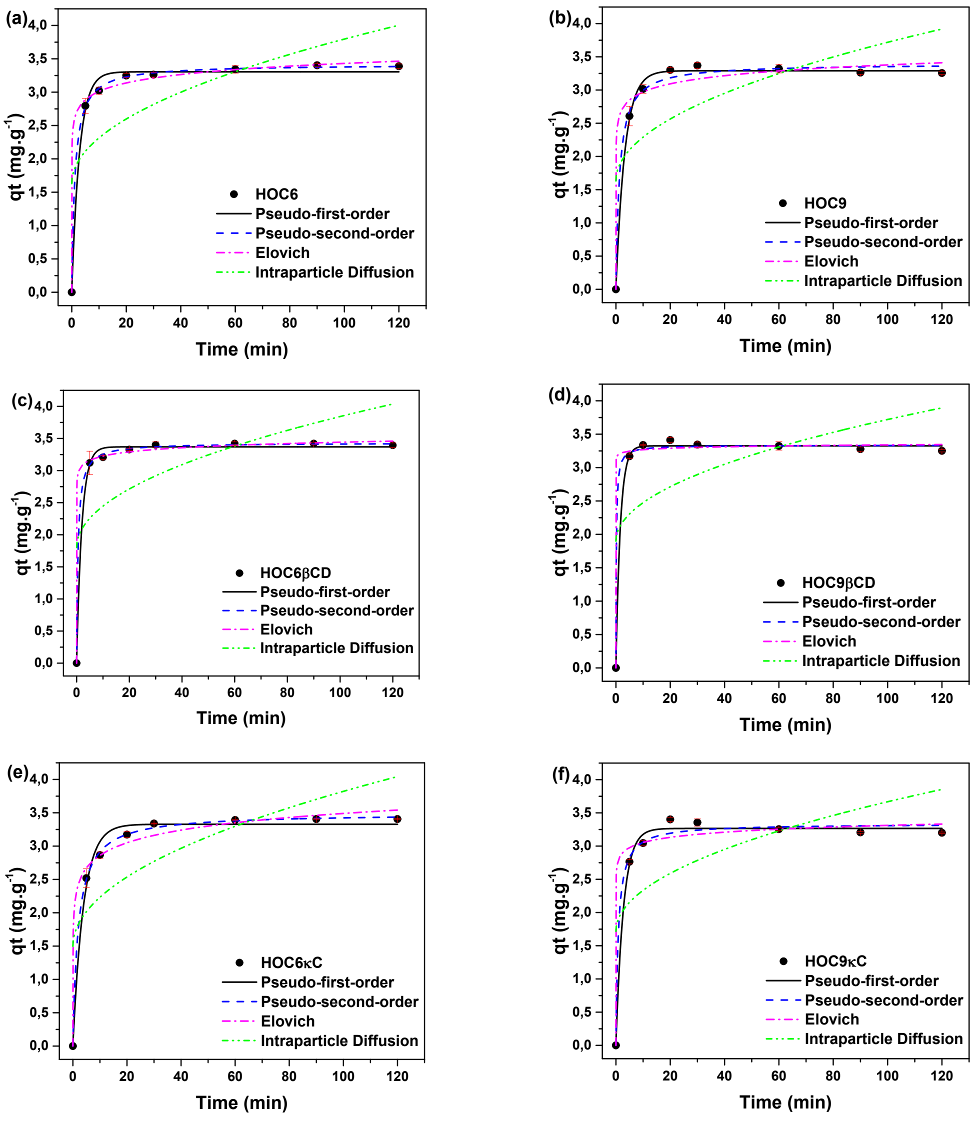

Fig. S1. Graphs of the fit by non-linear regression **(a-f)** of kinetic models applied to methylene blue adsorption kinetics for different adsorbents.

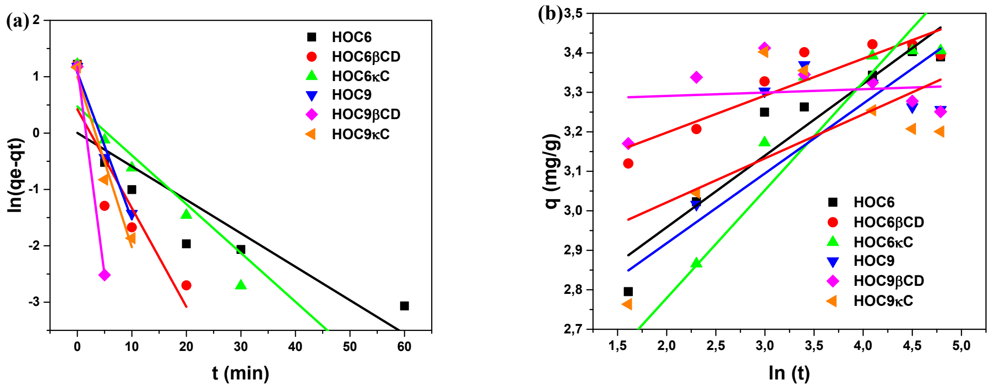

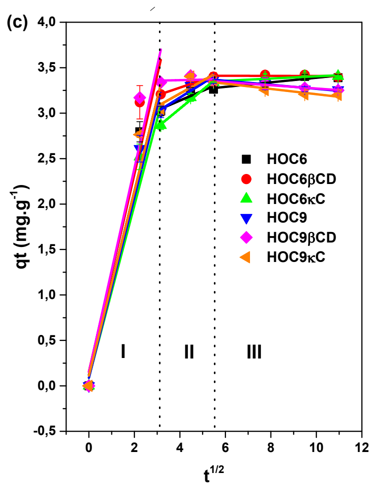

Fig. S2. Graphs of the fits by linear regression of the pseudo-first-order **(a)** Elovich **(b)** and intraparticle diffusion **(c)** kinetic models, applied to the adsorption kinetics of methylene blue for the different adsorbents.

The experimental data of adsorption equilibrium of the hydrogels at different MB concentrations were fitted by the two Langmuir and Freundlich isotherm models expressed in the equations (S8) e (S9), respectively.

$$q_e = \frac{q_{\max} K_L C_e}{1 + K_L C_e} \qquad \text{(S8)}$$

$$q_e = K_F C_e^{1/n} \qquad \text{(S8)}$$

where q_e (mg·g^{-1}) and C_e (mg·L^{-1}) are the equilibrium adsorption capacity and the equilibrium adsorbate concentration, respectively. $q_{\max}$ (mg·g^{-1}) and K_L (L·mg^{-1}) are constants of the Langmuir isotherm, representing the maximum adsorption capacity and the interaction constant between adsorbent and adsorbate, respectively. K_F ((mg·g^{-1})·(L·mg^{-1})$^{1/n}$) corresponds to the Freundlich constant related to the adsorption capacity of the adsorbent, and $1/n$ (dimensionless) represents the surface heterogeneity factor that reflects the adsorption intensity.

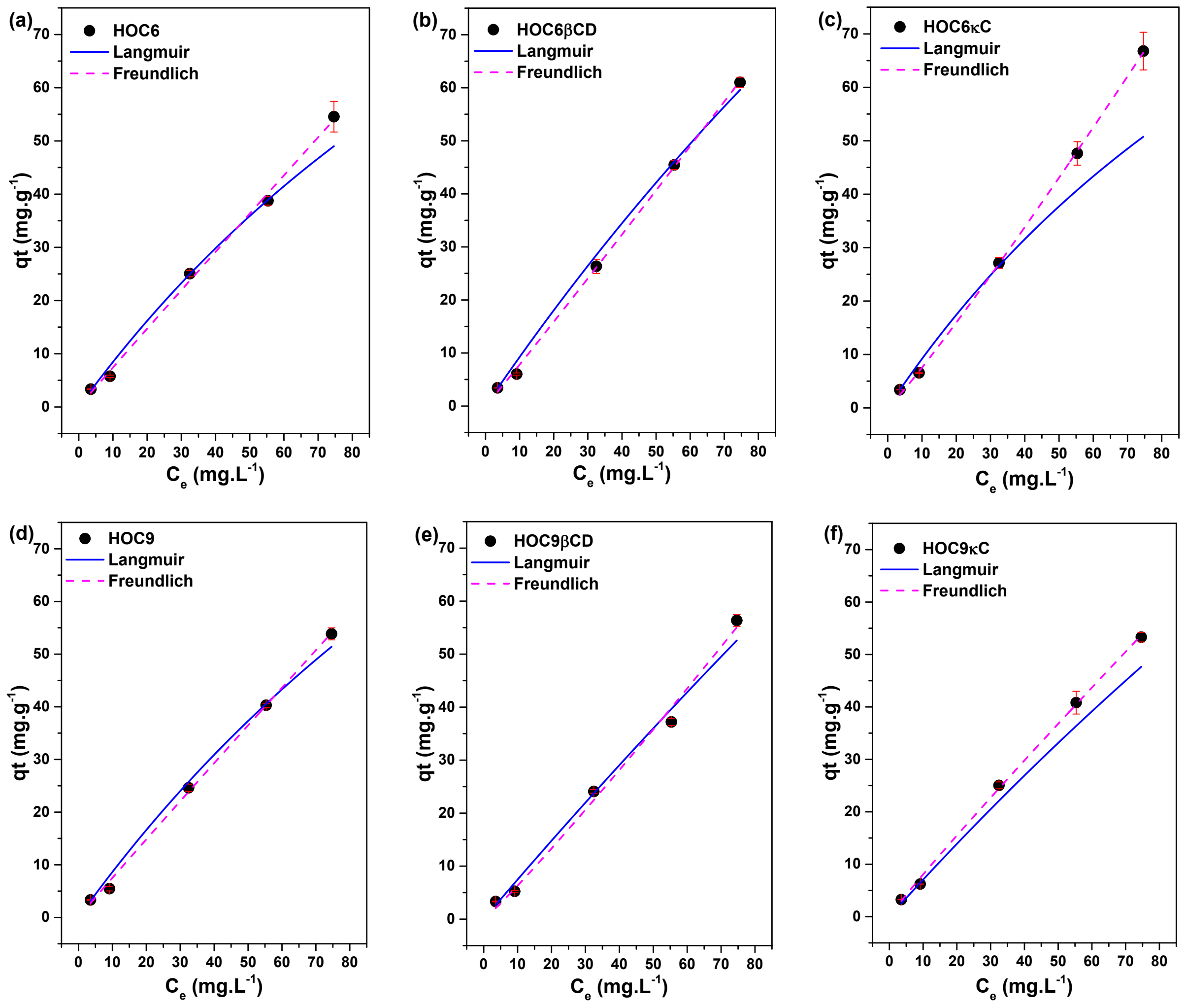

Fig. S3. Graphs of the fit by non-linear regression **(a-f)** of Freundlich and Langmuir isotherm models applied to methylene blue adsorption for different adsorbents.

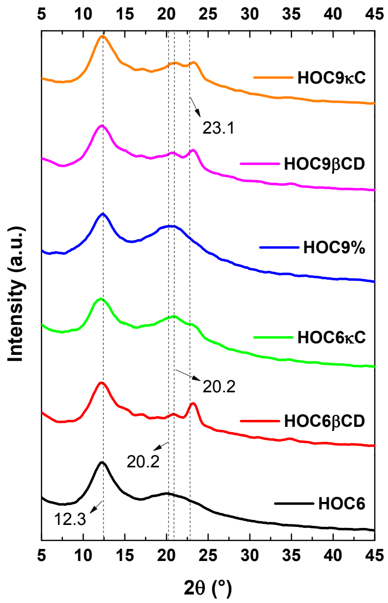

Fig. S4. XRD curves of hydrogels.
